# Rapid cycling as a feature of bipolar disorder and comorbid migraine

**DOI:** 10.1016/j.jad.2015.01.024

**Published:** 2015-04-01

**Authors:** K. Gordon-Smith, L. Forty, C. Chan, S. Knott, I. Jones, N. Craddock, L.A. Jones

**Affiliations:** a Institute of Psychological Medicine & Clinical Neurosciences, Cardiff University, UK; b Department of Psychiatry, School of Clinical and Experimental Medicine, University of Birmingham, UK; c Bipolar Disorder Research Network (BDRN, bdrn.org), UK

**Keywords:** Bipolar disorder, Migraine, Comorbidity, Rapid cycling

## Abstract

**Background:**

Previous research has suggested the clinical profile of individuals with bipolar disorder (BD) differs according to the presence or absence of comorbid migraine. We aimed to determine the clinical characteristics that differentiate individuals with BD with and without comorbid migraine in a large, representative, clinically well-characterised UK sample.

**Methods:**

The lifetime clinical characteristics of 1488 individuals with BD (BPI *n*=1120, BPII *n*=368) with and without comorbid migraine were compared (*n*=375 vs. *n*=1113 respectively).

**Results:**

Individuals with BD and comorbid migraine had a distinctive set of lifetime clinical characteristics. A multivariate model showed that consistent with previous studies those with comorbid migraine were significantly more likely to be female (OR=2.099, *p*=0.005) and have comorbid panic attacks (OR=1.842, *p*=0.004). A novel finding was that even after controlling for other differences, the individuals with BD and comorbid migraine were more likely to have a rapid cycling illness course (OR=1.888, *p*=0.002).

**Limitations:**

Presence of migraine was assessed using self report measures. Cross-sectional study design limits investigations of bidirectional associations between migraine and bipolar disorder.

**Conclusions:**

Comorbid migraine in BD may represent a more homogenous subtype of BD with an unstable rapid cycling course. Identifying individuals with BD and comorbid migraine may be of use in a clinical setting and this subgroup could be the focus of future aetiological studies.

## 1 Introduction

Migraine is a common chronic headache disorder, affecting approximately 12% of the general population (Breslau et al., 1991). The condition is characterised by recurrent attacks lasting 4–72 h, typically of a pulsating quality, moderate or severe intensity, aggravated by routine physical activity and associated with nausea, vomiting, photophobia or phonophobia (Headache Classification Subcommittee of the International Headache Society, 2004). Migraine is frequently comorbid with bipolar disorder (BD) (Low et al., 2003; Mahmood et al., 1999; McIntyre et al., 2006; Ortiz et al., 2010). Prevalence rates of migraine among individuals with BD have been reported to be around 25% (Mahmood et al., 1999; McIntyre et al., 2006; Ortiz et al., 2010). Although past studies have suggested that the prevalence of migraine is higher in affective disorders than in the general population with no difference between those with bipolar and unipolar disorder (Fasmer, 2001); one study found individuals with BD to be 2.9 times more likely to have migraine than those with major depressive disorder (Dilsaver et al., 2009) suggesting migraine is linked to bipolarity. A recent systematic review of BD-migraine comorbidity highlighted particularly high prevalence rates of migraine among women with bipolar II disorder (Fornaro et al., 2014).

Previous studies have shown that individuals with comorbid BD and migraine have different clinical characteristics to individuals with BD without migraine. Earlier age of onset of BD (Mahmood et al., 1999; McIntyre et al., 2006), increased prevalence of comorbid panic disorders (Fasmer and Oedegaard, 2001; Ortiz et al., 2010), higher prevalence of bipolar II disorder subtype (Fasmer and Oedegaard, 2001; Ortiz et al., 2010) and higher rate of attempted suicide (Ortiz et al., 2010) have all been associated with BD with comorbid migraine. These differences support the proposal that the presence of comorbid migraine represents a more homogenous subtype of BD. Clinical homogeneity may represent aetiological homogeneity and this subgroup could be the focus of future aetiological studies, which could lead to a better understanding of the pathogenesis of BD and migraine. Furthermore, this subgroup could benefit clinically from more effective targeted diagnostic and management strategies.

Further evidence is needed to confirm and refine BD and comorbid migraine as a suggested homogenous phenotypic subgroup. This study will build on previous smaller scale studies by examining differences in clinical characteristics between individuals with BD with and without comorbid migraine, using a large, representative, well-characterised UK nationwide sample.

## 2 Materials and methods

The study was part of our ongoing programme of research into the genetic and non-genetic determinants of BD and related mood disorders (Bipolar Disorder Research Network, BDRN; bdrn.org) which has UK National Health Service (NHS) Research Ethics Committee approval and local Research and Development approval in all participating NHS Trusts/Health Boards.

### 2.1 Recruitment of participants

Participants were recruited throughout the UK via systematic and non-systematic recruitment methods. Systematic recruitment involved screening for potential participants through Community Mental Health Teams and lithium clinics. Non-systematic recruitment involved advertisements for volunteers on the research team websites, in local and national media and through patient support organisations (such as Bipolar UK).

Our research programme inclusion criteria require participants to be aged 18 years and over, able to provide written informed consent and meet DSM-IV criteria for major affective disorder. Individuals are excluded if they: (i) experienced affective illness only as a result of alcohol or substance dependence or (ii) experienced affective illness only secondarily to medical illness or medication.

### 2.2 Psychiatric assessment

Participants were interviewed using the Schedules for Clinical Assessment in Neuropsychiatry (SCAN) (Wing et al., 1990), which provides detailed information about lifetime psychopathology. Psychiatric and general practice case-notes where available were also reviewed. Based on these data best-estimate lifetime diagnoses were made according to DSM-IV criteria and key clinical variables, such as age at onset and number of mood episodes, were rated. Level of function during each participant׳s lifetime worst episode of each of depression and (hypo)mania was assessed using the Global Assessment Scale (GAS) (Endicott et al., 1976). The GAS measures overall functioning of an individual during a specified time-frame on a continuum from illness to health with scores ranging from 1–100 (lowest–highest level of functioning). In cases where there was doubt, diagnostic and clinical ratings were made by at least two members of the research team blind to each other׳s rating and consensus was reached via discussion where necessary. Inter-rater reliability was formally assessed using 20 random cases. Mean kappa statistics were 0.85 for DSM-IV diagnoses and ranged between 0.81 and 0.99 for other key clinical categorical variables. Mean intra-class correlation coefficients were between 0.91 and 0.97 for key clinical continuous variables. Team members involved in the interview, rating and diagnostic procedures were all research psychologists or psychiatrists.

Participants in the current study (*n*=1488) were unrelated and met DSM-IV diagnostic criteria for bipolar I disorder (BPI) (*n*=1120) or bipolar II disorder (BPII) (*n*=368).

### 2.3 Migraine assessment and definition

History of migraine was assessed via two different methods during the course of our research programme.

#### 2.3.1 Self-rated migraine questionnaire

A self-rated questionnaire about lifetime experience of migraine consisted of 11 items relating to migraine frequency, character, duration, severity, associated symptoms, and aggravating and relieving factors. Participant responses were used to make a lifetime diagnosis of migraine according to criteria adapted from the International Headache Society diagnostic criteria.

#### 2.3.2 Doctor diagnosis of migraine

During a later wave of recruitment, information about lifetime history of migraine was assessed by an item on a self-report medical history questionnaire asking if participants had ever received a diagnosis of migraine headaches by a doctor.

Participants were stratified into the following two groups:

• Migraine group (*n*=375): participants with a lifetime diagnosis of migraine (*n*=118) according to the self-rated migraine questionnaire and participants who reported a doctor diagnosis of migraine (*n*=257).
• No migraine group (*n*=1113): participants who did not have a history of migraine according to the self-rated migraine questionnaire (*n*=211) or self-report medical questionnaire (*n*=902).

### 2.4 Statistical analysis

The data were analysed using SPSS. The majority of data were not normally distributed so non-parametric statistical tests were used. Demographic and clinical characteristics of the two groups were compared using chi-square tests for categorical data and Mann–Whitney U tests for continuous data. All demographic and clinical characteristics significant at *p*<0.05 in the univariate analyses were included as explanatory variables in a logistic regression model using the forward stepwise likelihood ratio method with absence or presence of migraine as the outcome/dependent variable.

## 3 Results

There was a significantly greater proportion of females in the migraine compared to no migraine group (80% vs. 67%, *p*<0.001). Individuals in the migraine group were significantly younger at interview (44 vs. 47 years, *p*=0.001). There were no other significant demographic differences between the migraine and no migraine groups (Table 1).

The clinical characteristics of BD in the migraine and no migraine groups are summarised in Table 2. Compared to participants without a history of migraines, individuals in the migraine group had significantly:

• Higher rate of history of panic attacks (63% vs. 44%, *p*<0.001)
• Higher rate of rapid cycling, defined as 4 or more episodes in any 12 month period (39% vs. 30%, *p*=0.011)
• Higher rate of family history of affective disorders (89% vs.83%, *p*=0.020)
• Lower rate of history of psychiatric admission (70% vs. 78%, *p*=0.002)
• Younger age at illness onset, defined as the age at which symptoms of affective disorder first caused significant impairment (19 vs. 21 years, *p*<0.001)
• Less impairment in functioning during worst episode of (hypo)mania, indicated by higher GAS scores (35 vs. 31, *p*=0.002)

Sex, age at interview and all clinical variables listed above were entered into a logistic regression analysis, which indicated the characteristics that best predicted the presence of comorbid migraine were being female (OR 2.099, 95% CI: 1.254–3.515, *p*=0.005), history of panic attacks (OR 1.842, 95% CI: 1.221–2.779 *p*=0.004) and a rapid cycling illness course (OR 1.888, 95% CI: 1.251–2.848, *p*=0.002).This model accounted 7.9% of the variance and correctly classified 79.2% of participants as having comorbid migraine or not.

## 4 Discussion

Our findings are in agreement with previous studies that have found differences in clinical characteristics between BD with and without comorbid migraine. The strength of our study is the large, robust, representative and clinically well-characterised sample. The prevalence of migraine in our sample was 25% which is accordant with previous studies (Mahmood et al., 1999; McIntyre et al., 2006; Ortiz et al., 2010).

When other significant differences were controlled for, individuals with comorbid migraine were more likely to be female, have comorbid panic attacks and a rapid cycling illness. Our finding of an independent association between rapid cycling BD and migraine, which remained significant in multivariate analysis, is novel. This has only been investigated in two other recent studies which also found a higher prevalence of rapid cycling among individuals with BD and comorbid migraine although this was not observed after correction for multiple comparisons (Brietzke et al., 2012b) or in multivariate analysis (Saunders et al., 2014). Brietzke et al (2012b) had a lower percentage of BPII cases than our BD sample (~10% vs. 25% respectively); however, as in our sample BPII disorder was not associated with migraine in their report. In the study by Saunders et al. (2014) lifetime serotonin noradrenaline reuptake inhibitor use was included in the multivariate model which was not included in the present study. The higher percentage of women in the migraine group is compatible with the higher rate of migraine among women than men in the general population (Breslau et al., 1991; Jette et al., 2008) and in previous studies in BD (Baptista et al., 2012; Saunders et al., 2014). In line with previous studies, comorbid migraine was associated with a higher prevalence of lifetime history of panic attacks (Fasmer and Oedegaard, 2001; Ortiz et al., 2010). Numerous studies have found a relationship between BD and panic disorder (Freeman et al., 2002) and we have previously found that the presence of panic attacks may differentiate between subgroups of individuals with BD (Forty et al., 2009). The findings of our current study suggest that the relationship between BD and panic attacks may be stronger among those with comorbid migraine. Individuals in the migraine group also had a significantly younger age of onset of BD.

In contrast to some previous studies that have found an association between migraine and BPII disorder (Fasmer and Oedegaard, 2001; Ortiz et al., 2010), we did not find a higher prevalence of BPII disorder in the migraine group. However, individuals in the migraine group had significantly higher GAS scores during their worst episode of high mood indicating less impairment in functioning in the univariate analyses. Because of previously reported associations between migraine and BPII disorder we repeated our analysis in the BPI and BPII groups separately and the outcomes were the same. Our findings in the whole BD sample were not accounted for by the BPII cases.

Our findings provide further evidence that comorbid migraine in BD represents a more homogenous subgroup of BD that may be associated with an unstable rapid cycling illness course. The identification of a distinct subgroup of individuals with BD has significant implications for future research and clinical practice. There is evidence that there are common neurobiological pathways underlying BD and migraine, for example, a number of pharmacological treatments including valproate are used to treat both conditions (Peterson and Naunton, 2005). Inflammatory cytokines have also been implicated in the neurobiology of both BD and migraine (Brietzke et al., 2012a). The aetiologies of both conditions are currently unknown although both disorders are recognised to have multifactorial polygenic aetiology and research suggests they may share common pathophysiology. A genome-wide association study of BD found evidence of association for several single-nucleotide polymorphisms on chromosome 13q14.1 with a small sample of patients with doctor-diagnosed comorbid migraine (*n*=56) (Oedegaard et al., 2010a). The role of ion channnelopathies has been proposed in the pathogenesis of both disorders and in particular calcium channelopathy. Genome-wide association studies have found evidence for an association between *CACNA1C* (alpha 1C subunit of the L-type voltage-gated calcium channel) gene variants and BD (Ferreira et al., 2008; Sklar et al., 2011) and mutations in a calcium channel encoding gene *CACNA1A* (alpha 1A subunit of P/Q type voltage-dependent calcium channel) have been found to cause familial hemiplegic migraine type 1 (Gargus, 2009). A genome-wide linkage study of BD and comorbid migraine identified an overlapping susceptibility locus for both disorders on chromosome 20p11, a region containing a gene involved in calcium homoeostasis, suggesting that some genes may predispose individuals to both BD and migraine (Oedegaard et al., 2010b). However, another study did not find any evidence of an association between a genetic variant in *CACNA1C* that has been associated with BD and migraine in a sample of 192 families with a proband with childhood migraine (Wober-Bingol et al., 2011).

A potential limitation of this study is that the presence/absence of migraine was determined by self-report. It is however reassuring that when the accuracy of the self-report medical history questionnaire was previously assessed, agreement between general practitioners and participants for the presence or absence of a medical disorder was 93% (Farmer et al., 2008). In order to increase our sample size, we combined data collected on migraine headaches during different waves of recruitment to our research programme. Analysis of the level of agreement between the two measures in a different sample found a moderate level of agreement (*k*=0.55) with the single migraine item from the medical history questionnaire being the more conservative of the two. We also carried out separate analyses on the participants who completed the migraine questionnaire (*n*=359) and the medical history questionnaire (*n*=1237) and the direction of association was the same in all cases, although the effect sizes were slightly smaller in the migraine questionnaire group. The amount of variance explained by the logistic regression was small (8%), although this was not unexpected given the multifactorial aetiology of migraine. For the majority of cases the presence of migraine was assessed by the single item on a medical questionnaire meaning we were unable to separate the migraine group further into those with and without aura. Further in depth analyses of comorbid migraine in BD are required in large samples with more detailed information about the migraine phenotype such as the presence of aura.

## Role of funding source

The funders (Wellcome Trust, and the Stanley Medical Research Institute) had no role in study design; in the collection, analysis and interpretation of data; in the writing of the report; or in the decision to submit the paper for publication.

## Conflict of interest

All authors report no conflict of interest.

## Figures and Tables

**Table 1:** Demographic characteristics according to diagnosis of migraine.

	Migraine (*n*=375)	No migraine (*n*=1113)	*P* value
**Sex,*****n*****(%)**			
Female	300 (80.0)	743 (66.8)	**<0.001**

**Age at interview (years)**			
Median	44	47	**0.001**
Iq R	16	20	
Range	19–73	18–80	

**Highest educational level,*****n*****(%)**			
CSE/O-level/GCSE	85 (23.5)	236 (22)	0.480
A-level/HND/BTEC	107 (29.6)	276 (25.7)	
Degree	95 (26.3)	316 (29.4)	
Postgraduate degree	46 (12.7)	154 (14.3)	

**Highest occupation level,*****n*****(%)**			
Legislators, senior officials, managers and professionals	123 (34.5)	407 (37.6)	0.063
Technicians, associate professionals and civil servants	63 (17.6)	202 (18.7)	
All other occupations	168 (47.1)	442 (40.9)	
Never worked	0 (0)	13 (1.2)	
Student	3 (0.8)	18 (1.7)	

**Recruitment,*****n*****(%)**			
Systematic	81 (22.2)	221 (20.1)	0.403

Iq R=interquartile range. Figures in bold indicate variables (*p*<0.05) to be entered into regression model. Totals vary due to unknown/missing data.

**Table 2:** Lifetime clinical characteristics of the migraine and no migraine groups.

	Migraine (*n*=375)	No migraine (*n*=1113)	*P* value
**DSMIV diagnosis,*****n*****(%)**			
BPI	280 (74.7)	840 (75.5)	0.755
BPII	95 (25.3)	273 (24.5)	
**History of panic attacks,*****n*****(%)**	154 (63.4)	392 (44.3)	**<0.001**
**History of rapid cycling,*****n*****(%)**	94 (38.8)	235 (30.1)	**0.011**
**History of suicide attempt,*****n*****(%)**	198 (54.1)	527 (49.3)	0.109
**Family history of affective disorder*****n*****(%)**	284 (88.8)	790 (83.3)	**0.020**
**History of psychotic symptoms,*****n*****(%)**	215 (66.8)	641 (66.8)	1.000
**History of psychiatric admission,*****n*****(%)**	257 (70.4)	848 (78.3)	**0.002**
**Age at illness onset**			
Median	19	21	**<0.001**
Iq R	10	12	
Range	4–63	4–64	
**No. episodes depression**			
Median	8	8	0.232
Iq R	16	16	
Range	0–250	0–100	
**No. episodes (hypo)mania**			
Median	6	6	0.509
Iq R	8	8	
Range	1–250	1–100	
**GAS score during worst depression**			
Median	40	40	0.489
Iq R	14	13	
Range	10–61	10–71	
**GAS score during worst (hypo) mania**			
Median	35	31	**0.002**
Iq R	30	28	
Range	10–61	8–68	

Iq R=inter-quartile range; GAS=Global Assessment Scale; BPI=bipolar I disorder; BPII=bipolar II disorder. Figures in bold indicate variables (*p*<0.05) to be entered into regression model. Totals vary due to unknown/missing data.
